# Biotech Application of Exopolysaccharides from *Curvularia brachyspora*: Optimization of Production, Structural Characterization, and Biological Activity

**DOI:** 10.3390/molecules28114356

**Published:** 2023-05-26

**Authors:** Rafael Andrade Menolli, Fernando Henrique Galvão Tessaro, Alex Evangelista do Amaral, Renan Henrique de Melo, Jean Felipe dos Santos, Marcello Iacomini, Fhernanda Ribeiro Smiderle, Rosiane Guetter Mello

**Affiliations:** 1 Center of Medical and Pharmaceutical Sciences, Western Parana State University, Cascavel 85819-110, PR, Brazil; renan_henriquedemelo@hotmail.com; 2 Faculdades Pequeno Príncipe, Curitiba 80230-020, PR, Brazil; jean.fes@hotmail.com (J.F.d.S.); rosiane.mello@fpp.edu.br (R.G.M.); 3 Instituto de Pesquisa Pelé Pequeno Príncipe, Curitiba 80240-020, PR, Brazil; 4 Faculdade de Ciências Farmacêuticas, Universidade de São Paulo, São Paulo 05508-000, SP, Brazil; fernando.tessaro@gmail.com; 5 Unidade de Laboratório de Análises Clínicas, Universidade Federal de Santa Catarina, Florianópolis 88036-800, SC, Brazil; alex.amaral@ufsc.br; 6 Department of Biochemistry and Molecular Biology, Federal University of Paraná, Curitiba 81531-980, PR, Brazil

**Keywords:** dematiaceous fungi, response surface methodology, polysaccharide, fungal production, biological activity

## Abstract

*C. brachyspora*, a widespread dematiaceous fungus, was evaluated in this study to optimize the production of exopolysaccharides (CB-EPS). Optimization was performed using response surface methodology, and the best production yielded 75.05% of total sugar at pH 7.4, with 0.1% urea, after 197 h. The obtained CB-EPS showed typical signals of polysaccharides, which was confirmed by FT-IR and NMR. The HPSEC analysis indicated a polydisperse polymer, showing a non-uniform peak, with an average molar mass (M*_w_*) of 24,470 g/mol. The major monosaccharide was glucose (63.9 Mol%), followed by mannose (19.7 Mol%), and galactose (16.4 Mol%). Methylation analysis encountered derivatives that indicated the presence of a β-d-glucan and a highly branched glucogalactomannan. CB-EPS was tested on murine macrophages to verify its immunoactivity, and the treated cells were able to produce TNF-α, IL-6, and IL-10. However, the cells did not produce superoxide anions or nitric oxide nor stimulated phagocytosis. The results demonstrated an indirect antimicrobial activity of macrophages by stimulating cytokines, showing another biotech applicability for the exopolysaccharides produced by *C. brachyspora*.

## 1. Introduction

Polysaccharides are an important class of bioactive substances and constitute a significant proportion of fungal biomass [1]. Fungal polysaccharides are mainly utilized in the food and pharmaceutical industries due to their important applicability, such as physicochemical modifiers and biological activities. Various chemical characteristics, such as specific linkage types, high molecular weight, and heteropolymeric composition, affect polysaccharides [2]. In addition, these macromolecules are relatively non-toxic to normal cells and tissues, which affords no significant side effects if used as therapeutic drugs [3]. Based on such evidence, many studies about fungal polysaccharides, their sources, and how to produce them have been published, showing the potential of biotechnological tools to obtain bioactive compounds [1,2,3,4,5]. Among the biological properties described for such macromolecules, it can be mentioned: antioxidant, anti-inflammatory, antiangiogenic, antiviral, antitumor, and immunomodulatory activities [6,7,8].

Since microscopic fungi produce and excrete different polysaccharides, fungal exopolysaccharides (EPS) present advantages compared to intracellular ones, such as easier isolation and purification processes [9]. Therefore, researchers have extensively investigated fungal EPS regarding their production, chemical structures, and biological activities to find and produce ideal compounds for developing novel pharmaceutical agents [8].

The dematiaceous (brown-pigmented) fungi are a large and heterogeneous group of filamentous fungi that rarely cause human infections, being poorly explored as EPS producers. A member of this group is *Curvularia brachyspora*, a black mold with its biotech capability recently tested in feather and petroleum degrading [10,11]. It is also classified as a fungal endophyte in stressful habitats plants [12] and a producer of organic compounds such as oxanes [13]. However, in-depth knowledge about this species and its potential as a bioactive compound producer is still absent in scientific literature.

Therefore, this study demonstrated for the first time the production and optimization of EPS from *C. brachyspora* and its chemical characterization and biological activities on macrophages.

## 2. Results and Discussion

### 2.1. Model Adjustment and Optimization of Production

*C. brachyspora* maximized its production of exopolysaccharides using submerged cultivation from 17 combinations of three independent variables pH (X_1_), incubation time (X_2_), and urea concentration (X_3_) (Table 1).

The percentage of total sugars in exopolysaccharides ranged between 7.26 and 75.05%, showing that the RSM can provide a considerable variation in the results, with few experiments combining the variables correctly.

The response surface methodology yielded the following regression equation, which is an empirical relationship between the production of exopolysaccharide and the tested variables coded as shown in Equation (1):

(1) $$\begin{array}{l} Y=32.50884\;-7.99052x_{1}+8.21789x_{2}-3.16826x_{3}-2.01170x_{1}^{2}\\ +1.96516x_{2}^{2}-1.70762x_{3}^{2}-7.19375x_{1}x_{2}+7.06440x_{2}x_{3}-4.66892x_{1}x_{3} \end{array}$$

A positive sign in front of the terms indicates a synergistic effect, while a negative sign represents an antagonistic effect toward the percentage of total sugar in EPS. Thus, from the equation, the rate of total sugar in EPS will increase with the variable X_2_ (time) in primary and quadratic effect and will decrease with the other two (X_1_ and X_3_—pH and urea) in the same way, while the interactions X_1_X_2_ and X_1_X_3_ cause a decrease of production, the interaction X_2_X_3_ provoked an increase.

The analysis of variance (ANOVA) of independent variables and their interactions is shown in Table 2.

The total determination coefficient (R^2^) was 89.74, indicating a good fit for experimental data. Furthermore, the model showed that the calculated F (6.80) is greater than the tabulated F (3.68), and all these values, together with R^2^ and *p* < 0.05 indicate a strong correlation between the theoretical model and experimental data with a significance at a 95% confidence level.

Two preliminary factorial experiments (Supplementary Tables S1 and S2) have been completed to decide the parameters that would be optimized: firstly, the physical parameters were refined, while the second step evaluated the sources of carbon and nitrogen. The literature reports that micro-components, such as salts or vitamins, have rarely influenced EPS production, with the parameters of pH, temperature, time, C and N sources being the most important [14].

The maximum yield was reached in run 3 (Table 1), under pH 7.4, 197 h, and 0.1% of urea, with 75.05% of total sugar in EPS, which is higher than achieved in the first factorial (23.9%), in which only physical variables were evaluated with basal medium (inorganic salts and glucose, Table S1). Higher values are reached on the second factorial (70.50%) (Table S2), with similar values to those achieved in the DCCR experiment, which was completed to reach a quadratic function with an optimized condition.

The significance of each model towards the production of EPS could be ranked according to their F values and *p*-values: the incubation time (X_2_) showed the highest effect, while the urea concentration (X_3_) presented the least among the primary effects. The quadratic effects had no significance in the production, following the worst F values between all terms.

The variables pH (X_1_), incubation time (X_2_) alone, as well as the interactions between pH and incubation time (X_1_X_2_), and pH and urea concentration (X_1_X_3_), showed statistical significance in EPS production, as observed in Table 2.

The incubation time presented the most significant influence on EPS production by *C. brachyspora* compared with the other two variables. Usually, time is essential in the optimization process using mycelial fungi [15] and positively influenced the present study. Initial pH was the second most crucial variable, negatively influencing the obtainment of total sugar after the growth, demonstrating that the initial pH is one of the most crucial growth conditions for anemophilous fungus [16,17]. Nitrogen supplementation is another variable that several studies reported to induce EPS production. Among the inorganic sources, ammonium chloride, ammonium sulfate, sodium nitrate, potassium nitrate, urea, and diammonium oxalate monohydrate are commonly used [14]. Here, urea (0.1%) was the most effective compound as a nitrogen source, surpassing ammonium nitrate and sodium nitrate, similar to an optimized EPS production by *Nigrospora oryzae* [18]. In general, little nitrogen is required by fungi for EPS production, and concentrations between 0.1–1% are sufficient [14].

One of the advantages of using RSM to study the effect of experiment variables compared to conventional methods is that it considers the possible consequences of the variables. The interactions between variables can be illustrated in three-dimensional graphics, showing the main interactive effects of independent variables on the dependent. The software generates the graphs by setting a zero-coded variable (Table 1), varying the other two variables to predict response variables (sugar yield).

**Figure 1:**
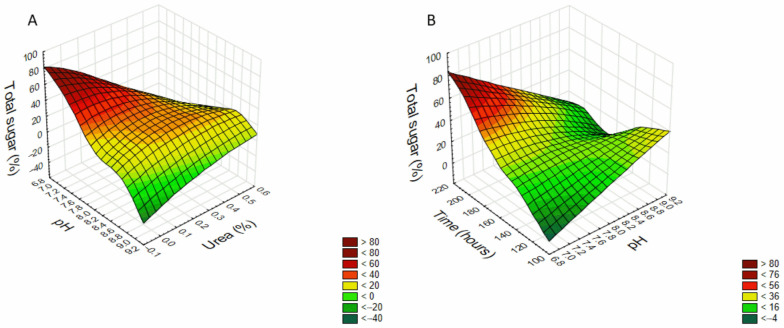
Three-dimensional response surface graph showing the effect of (**A**) pH and Urea (time fixed at 168 h—central point) and (**B**) pH and time (Urea concentration fixed at 0.25%—central point) in the exopolysaccharide production by *C. brachyspora*.

Figure 1 shows no quadratic effect in the interaction of pH and urea (%) (A) and pH and time (hours) (B) in EPS production, and, according to Equation (1), a decrease in pH and urea concentration (Figure 1A), and an increase in incubation time with a decrease in the pH (Figure 1B) enhances the production of the exopolysaccharides. The figures also show a higher production scaling up the surface, i.e., decreasing urea and pH (A) and increasing incubation time and decreasing pH (B). However, after performing production kinetics at the best condition, pH 7.4 and 0.1% urea (Figure S1), it was observed that the glucose medium was exhausted on day ten, and the EPS production consequently reduced at this time point.

### 2.2. Chemical Characterization of Exopolysaccharides Produced by C. brachyspora

**Figure 2:**
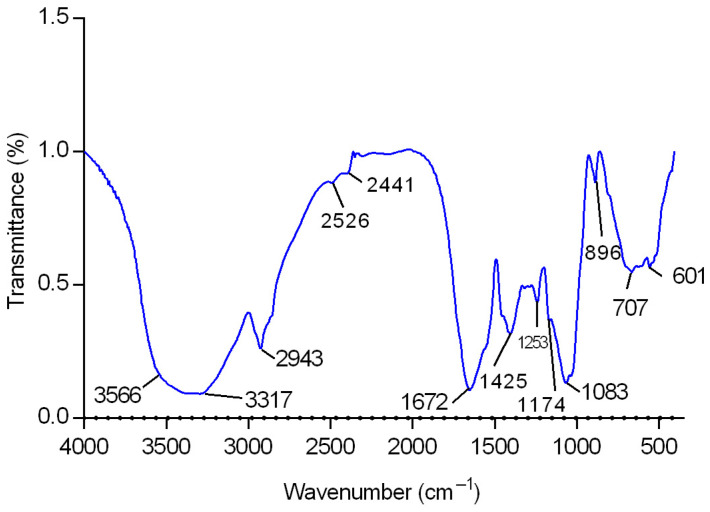
FT-IR spectrum of sample CB-EPS in the 4000–400 cm^−1^ region.

After determining the best growth condition of the fungus to produce EPS, the chemical characterization of such macromolecules was performed. The exopolysaccharides (CB-EPS) from *C. brachyspora* were recovered as previously described (Section 3.5). This fraction was analyzed by infrared spectroscopy (FT-IR), and it showed high absorbance at wave numbers characteristic of sugars (Figure 2), confirming the presence of polysaccharides. On the ‘sugar region’ (950–1200 cm^−1^), it was observed C-O stretching of pyranose rings at 1083 cm^−1^ [19]; while the absorbance at 896 cm^−1^ confirmed C-O-C stretching of glycosidic bonds, typical of fungal glucans in β-configuration [19,20,21]. Other peaks could be assigned such as C=O groups at 1672 cm^−1^; C-H stretching at 2943 cm^−1^; and an intense broadband at the range 3317–3566 cm^−1^ that indicated hydroxyl group [20,22].

The monosaccharide composition was determined by gas chromatography (Figure S2), and the CB-EPS presented glucose (63.9%), mannose (19.7%), and galactose (16.4%).

**Figure 3:**
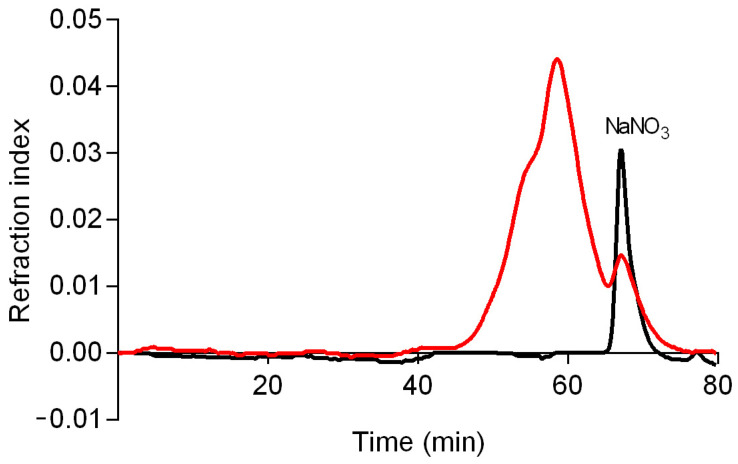
Elution profile of CB-EPS (red) and eluent alone (black) determined by HPSEC using refractive index detectors. The eluent was 0.1 NaNO_3_ and the sample was previously solubilized in the eluent at 1 mg/mL.

A high-performance size exclusion chromatography (HPSEC) analysis (Figure 3) demonstrated that CB-EPS is a polydisperse polymer, showing a non-uniform peak, which can be observed by the two merged peaks, starting at 45 min and ending at 65 min. This fraction presented an average molar mass (M*_w_*) of 24,470 g/mol (with an error margin of 5%). M*_w_* was calculated with the software Astra (version 4.70) using as ∂n/∂c value of 0.114 mL/g [23].

**Figure 4:**
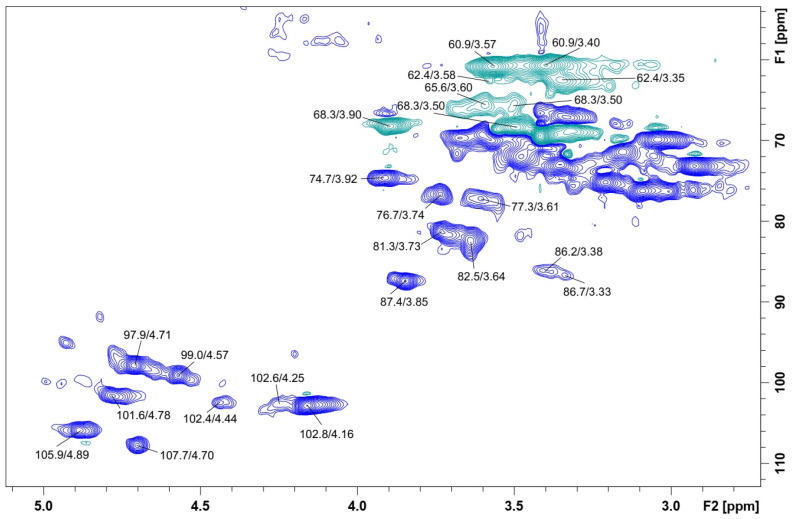
HSQC-DEPT spectrum of CB-EPS obtained from submerged culture of *C. brachyspora*. Sample was solubilized in Me_2_SO-*d*_6_ and the analysis was performed at 70 °C (chemical shifts are expressed in ppm).

NMR analysis (HSQC-DEPT) confirmed the results observed on FT-IR showing signals of polysaccharides from 50.0 to 110.0 ppm for ^13^C and from 2.80 to 5.00 ppm for ^1^H (Figure 4). The main signals were determined by correlating the NMR data and comparing the literature values of similar polysaccharides. The spectrum confirmed the presence of glucopyranose, mannopyranose, and galactofuranose as major monosaccharides by the signals in the anomeric region at δ 102.4/4.44, 102.6/4.25 and 102.8/4.16 (β-Glc*p* units); at δ 101.6/4.78, 99.0/4.57, and 97.9/4.71 (α-Man*p*); at δ 107.7/4.70 and 105.9/4.89 (β-Gal*f* units).

Furthermore, a methylation reaction was performed to determine the linkages of CB-EPS and it was possible to correlate some NMR signals with *O*-linked derivatives observed on methylation analysis. The results identified two polysaccharides commonly isolated from Ascomycetes fungi: a β-d-glucan and a glucogalactomannan. The first polysaccharide was confirmed by the presence of 2,3,4,6-Me_4_-Glc*p*, 2,4,6-Me_3_-Glc*p*, and 2,4-Me_2_-Glc*p* derivatives (Table 3); and the NMR signals at δ 86.2/3.38 and 86.7/3.33 confirmed the presence of *O*-substituted C3/H3 of 1,3- and 1,3,6-linked β-Glc*p* units [24,25], while the signals at δ 68.3/3.90; 3.50 confirmed the *O*-6-substitution of 1,3,6-linked units [24,25]. The heteropolysaccharide showed a higher complexity, with the following methyl derivatives: 2,3,4,6-Me_4_-Glc*p*, 2,3,5,6-Me_4_-Gal*f*, 2,3,4,6-Me_4_-Gal*p*, 3,4,6-Me_3_-Man*p*, 2,3,4-Me_3_-Man*p*, 2,3,6-Me_3_-Gal*f*, 2,3,5-Me_3_-Gal*f*, 3,4-Me_2_-Man*p*, and 3,5-Me_2_-Gal*f* (Table 3).

Some correspondent NMR signals could be observed at δ 77.3/3.61 and δ 65.6/3.60 and 3.50, which confirmed the glycosylation sites (C2/H2 and C6/H6, respectively) of 6- and 2,6-linked-mannose [23,26]. Resonances relative to *O*-2-, *O*-5- and *O*-6-substitutions in galactofuranose units were seen at δ 87.4/3.85, δ 76.7/3.74, and δ 72.0/3.48, respectively [23,25,26,27,28,29,30,31]. Other resonances observed are described in Table 4.

The presence of 3,4-Me_2_-Man*p* and 3,5-Me_2_-Gal*f* and the tremendous amount of non-reducing ends (Table 3) indicate that the glucogalactomannan is highly branched. According to the results, the putative main chain comprises mannopyranose units *O*-2- and *O*-6-linked, with side branches containing galactofuranose linked at *O*-2, *O*-5- and/or *O*-6-linked with terminal glucopyranose units. Similar structures have been characterized for *Cordyceps militaris* [32], *Exophiala jeanselmei* [23], *Plectosphaerella cucumerina*, and *Verticillium* spp. [33], *Peltigera canina* [34], and *Clonostachys rosea* [30].

**Table 4:** ^1^H and ^13^C NMR chemical shifts of CB-EPS produced by *C. brachyspora*. Assignments are based on HSQC-DEPT analysis.

Units	^13^C Sign ^a^	^1^H Sign ^a^	References
C1/H1 of β-d-Gal*f*-(1→	107.7	4.70	[26,32]
C1/H1 of →X)-β-d-Gal*f*-(1→ ^b^	105.9	4.89	[26]
C1/H1 of β-d-Glc*p*-(1→	102.8	4.16	[24,25]
C1/H1 of →3)-β-d-Glc*p*-(1→	102.6	4.25	[24,25]
C1/H1 of →3,6)-β-d-Glc*p*-(1→	102.4	4.44	[24,25]
C1/H1 of →2)-α-d-Man*p*-(1→	101.6	4.78	[30]
C1/H1 of →6)-α-d-Man*p*-(1→	99.0	4.57	[30]
C1/H1 of →2,6)-α-d-Man*p*-(1→	97.9	4.71	[30]
C2/H2 of →2,6)-β-d-Gal*f*-(1→	87.4	3.85	[27]
C3/H3 of →3)-β-d-Glc*p*-(1→ or →3,6)-β-d-Glc*p*-(1→	86.7	3.33	[24,25]
C3/H3 of →3)-β-d-Glc*p*-(1→ or →3,6)-β-d-Glc*p*-(1→	86.2	3.38	[24,25]
C2/H2 of β-d-Gal*f*-(1→	82.5	3.64	[26]
C2/H2 of →2)-α-d-Man*p*-(1→ or →2,6)-α-d-Man*p*-(1→	77.3	3.61	[32]
C2/H2 of →2)-α-d-Man*p*-(1→ or →2,6)-α-d-Man*p*-(1→	76.7	3.74	[32]
C5/H5 of →5)-β-d-Gal*f*-(1→	74.7	3.92	[35]
C6/H6 of →3,6)-β-d-Glc*p*-(1→	68.3 ^c^	3.90/3.50	[24,25]
C6/H6 of →6)-α-d-Man*p*-(1→ or →2,6)-α-d-Man*p*-(1→	65.6 ^c^	3.60/3.50	[32]
C6/H6 of β-d-Gal*f*-(1→	62.4 ^c^	3.58/3.35	[23,26]
C6/H6 of →3)-β-d-Glc*p*-(1→	60.9 ^c^	3.57/3.40	[24,25]

^a^ The chemical shifts are expressed as ppm (δ); ^b^ Letter X represents: *O*-2, *O*-5 and/or *O*-2,6-substittions.; ^c^ Inverted signals in the DEPT experiment.

### 2.3. Effects of CB-EPS on Macrophage Activity and Viability

**Figure 5:**
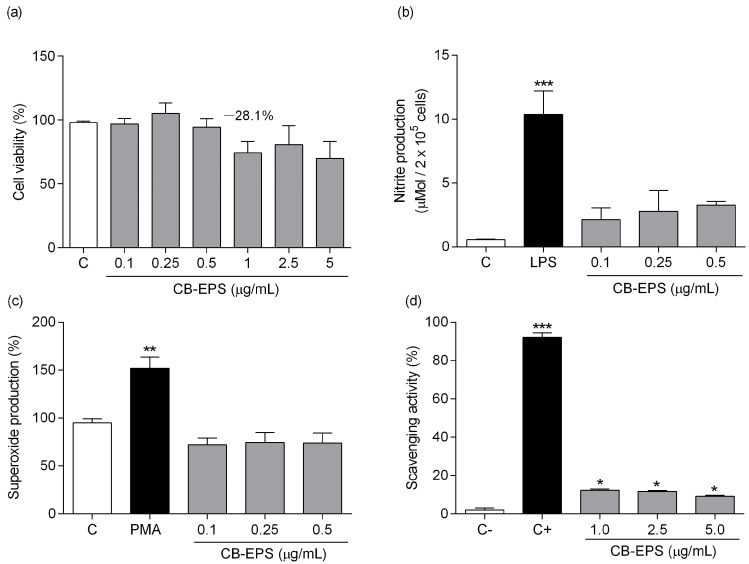
Effects of CB-EPS on macrophage viability (**a**), nitric oxide production (**b**), superoxide production (**c**), and scavenging activity (**d**). The values shown are the mean ± SEM of three independent experiments performed in triplicate. * *p* < 0.05; ** *p* < 0.01; *** *p* < 0.001, in comparison to the control. Control in (**a**–**c**) corresponds to the medium without CB-EPS. C+ in (**d**) corresponds to a reaction in which no superoxide production occurred, and C− corresponds to a reaction with superoxide production without any inhibitor.

The effects of the CB-EPS on the viability of murine peritoneal macrophages were evaluated after 48 h of treatment with variable polysaccharide concentrations (Figure 5a). Concentrations above 1 µg/mL resulted in a plateau of cytotoxicity, promoting nearly 70% cell death compared to the control group (medium). Therefore, subsequent experiments on cells held a concentration below 1 μg/mL.

Production of NO by murine peritoneal macrophages was determined after the same incubation period with CB-EPS (Figure 5b) and showed a tendency to increase slightly, but the results were not significant. A significant increase was observed at 1 μg/mL; however, it could be related to the cell death that was induced at this concentration, according to the viability test (Figure 5a). In addition, CB-EPS’s ability to induce superoxide production in macrophages was also tested (Figure 5c). It was observed no induction of such radical, unlike the positive control with PMA. The scavenging ability (Figure 5d) of CB-EPS was detected at significant levels, showing similar activity for the three concentrations tested (12.4%, 11.8%, and 9.2%). The results indicate that CB-EPS do not cause harm to murine peritoneal macrophages at concentrations equal to or below 0.5 μg/mL and do not stimulate the production of reactive oxygen/nitrogen species, but they induce a small scavenging activity in vitro.

**Figure 6:**
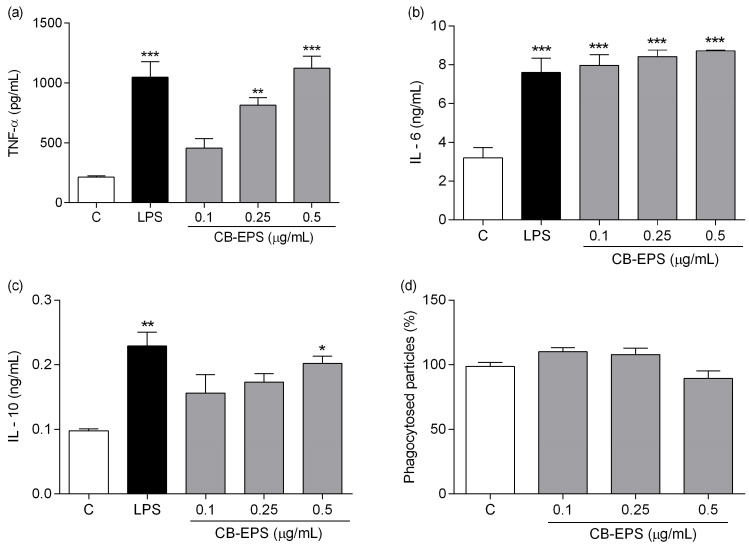
Effects of CB-EPS on macrophage production of TNF-α (**a**), IL-6 (**b**), IL-10 (**c**), and phagocytosis (**d**). The results are expressed as the mean ± SEM of three independent experiments performed in triplicate. * *p* < 0.05; ** *p* < 0.01; *** *p* < 0.001, in comparison to the control. Control corresponds to the medium in the absence of CB-EPS.

The immunomodulatory effect of CB-EPS was evaluated by measuring the secretion of TNF-α, IL-6, and IL-10 in supernatants of peritoneal macrophage cultures treated for six hours (for TNF-α evaluation) and 48 h (for IL-10 and IL-6 measurements). Concentrations of 0.25 and 0.5 μg/mL significantly increased TNF-α production (Figure 6a), and although the lower concentration did not show significant induction of TNF-α, the result shows a tendency to act as dose-dependent production of this cytokine with increasing concentrations of CB-EPS. As shown in Figure 6b, CB-EPS significantly increased the level of IL-6 in all concentrations, compared with the control group, reaching levels, such as those achieved by cells stimulated with LPS. No differences among the concentrations used were detected.

Finally, after incubating cells with CB-EPS for 48 h, peritoneal macrophages significantly increased the production of IL-10 at a higher concentration (Figure 6c). Indeed, the production was similar to that obtained by LPS-stimulated macrophages. In contrast with the result observed about cytokine secretion by the macrophages, phagocytosis was not stimulated after treatment with the exopolysaccharides (Figure 6d).

The macrophages express surface pattern recognition receptors (PRRs), which are targets of various ligands, including specific polysaccharides [36,37]. The ligand-bound receptor complexes can trigger the production and release of a wide range of mediators, including ROS and NO [38] and pro- and anti-inflammatory cytokines [39]. TNF-α and IL-6 are, among other pro-inflammatory cytokines, critical for the defense effect of peritoneal macrophages, while IL-10, a suppressive cytokine, plays an inhibitory function in inflammation [39,40]. CB-EPS showed the capacity to stimulate the production of pro- and anti-inflammatory cytokines; however, it did not induce the liberation of reactive oxygen/nitrogen species. This result indicates an indirect antimicrobial mechanism observed on macrophages, without the production of ROS/NO, but by secreting cytokines, which help to orchestrate the subsequent innate and adaptive immune responses [41].

## 3. Materials and Methods

### 3.1. Microorganism

*C. brachyspora* was obtained from the culture collection of the Federal University of Pernambuco, Recife—PE, Brazil (mycology collection URM/UFPE) number URM—3826. *C. brachyspora* stock culture was maintained on potato-dextrose-agar (PDA), incubated at 28 °C for seven days, stored at 8 °C, and subcultured every four weeks. The pre-inoculum was prepared by adding 2.5 mL of sterile water to the stock culture in PDA, followed by gentle scraping to obtain the spores. PDA was obtained from MilliporeSigma, St. Louis, MO, USA.

### 3.2. Conditions of Culture and Optimization of EPS Production

*C. brachyspora* was inoculated in 250 mL Erlenmeyer flasks containing 50 mL of a primary medium consisting of K_2_PO_4_ (0.1%), KH_2_PO_4_ (0.046%), MgSO_4_.7H_2_O (0.05%), and distilled water q.s.p. Glucose and nitrogen sources were added following the optimization schedule. All chemicals used in this procedure were obtained from MilliporeSigma, USA.

### 3.3. Design of Statistical Experiments

Response surface methodology was used to determine the influence of three independent variables and the optimum conditions of crude polysaccharide production. Preliminary determination of the variables used for exopolysaccharide production by *C. brachyspora* was completed in two experiments using factorial design. The first factorial was a 2^4^ experimental design, with 19 runs, using as variables: pH, temperature (°C), shaking (RPM), and time (days), which levels and values are in Table S1 (Supplementary Material). The second factorial experiment was a 2^6−1^ experimental design, with 35 runs, using as variables: Glucose concentration (%), pH, time (days), Ammonium Nitrate concentration (%), Urea concentration (%), Sodium Nitrate concentration (%). The levels and values of this experiment are in Table S2 (Supplementary Material). These two factorials provided as best culture conditions: glucose concentration of 1%, agitation at 90 rpm, and temperature of 28 °C, while the best nitrogen source observed was urea. Finally, we started a Central Composite Rotational Design with three independent variables (X_1_, initial pH; X_2_, incubation time (hours); X_3_, urea concentration), which was performed with five levels [42]. The values of the independent variables and their levels in a complete experimental design are shown in Table 1, in which the values of the percentage of total sugar existent in exopolysaccharide (mg) obtained in each run are the dependent variable. The DCCR consisted of 17 experimental points (eight factorial points, six axial points, and three central points), and the experiment was carried out randomly. The data DCCR were analyzed by multiple regression to fit a quadratic polynomial model using STATISTICA 7.0 software.

The surface plots were generated by assigning constant values to the three variables and solving the equations set as a quadratic equation in the one remaining variable. The significance of each coefficient was determined using the *F*-test and *p*-value. The corresponding variables would be more significant if the absolute *F*-value becomes higher and the *p*-value becomes smaller [43].

### 3.4. Polysaccharide Production and Purification

To obtain the exopolysaccharides after the growth of *C. brachyspora*, the media with the mycelial mass was harvested by filtration through a filter paper, and the filtrates were added to excess ethanol (3:1; *v*/*v*) and left overnight at 4 °C. The EPS was recovered after centrifugation (4500× *g* for 15 min at 25 °C), and the resulting polysaccharide precipitate was submitted to a rotary evaporator to eliminate the ethanol, and the residue was dialyzed against distilled H_2_O to remove low molecular-weight material and frozen. The frozen material was allowed to thaw slowly. Then, cold water-insoluble materials were centrifuged-off (4500 rpm for 20 min at 25 °C), and soluble materials were freeze-dried. After this, the soluble fraction, named CB-EPS, was used to determine the total sugar content. This procedure was performed on a large scale to obtain the CB-EPS, which is the exopolysaccharide obtained from the *C. brachyspora* growth at the optimized condition and used in this study’s chemical analyses and biological activities.

### 3.5. Determination of Total Sugar

The phenol-sulfuric acid method quantified the total sugar content in EPS [44], and glucose was used as the standard. The CB-EPS was weighted (mg/dL), and the total sugar determined in its composition was expressed as the content percentage. Phenol-sulfuric acid was obtained from MilliporeSigma, USA.

### 3.6. Monosaccharide Composition

CB-EPS fraction (1 mg) was hydrolyzed with 2 M TFA at 100 °C for 8 h, followed by evaporation to dryness, with nitrogen flow and resuspended in H_2_O (1 mL). The reduction was performed with NaBH_4_ (2 mg), at room temperature for 14 h, then the samples were treated with cationic resin. The solution was evaporated to dryness and washed thrice with methanol to remove boric acid. Acetylation was performed with acetic anhydride-pyridine (200 µL; *v*/*v*) at room temperature for 14 h.

The resulting alditol acetates were extracted with CHCl_3_ and analyzed by gas chromatography-mass spectrometer (GC-MS) using a single-quadrupole apparatus, model QP2020 (Shimadzu), equipped with an AOC-6000 autosampler (Pal Systems), operating in liquid injection mode. The sample was prepared in 1 mL of acetone and 1 µL was injected in split mode (1:10), at 250 °C. The chromatography was developed in a capillary column, RTX-5-MS (30 m × 0.25 mm × 0.25 µm film thickness). Helium (5.0 analytical grade) was used as a carrier gas with a linear velocity of 45 cm/s, at a flow rate of 1.54 mL/min. The oven was programmed with a non-linear heat ramp, starting at 50 °C and holding for 1 min, increasing to 150 °C at a range of 20 °C/min, and then reaching 200 °C at a range of 5 °C/min, being held for 3 min. The final temperature was set at 250 °C, heated at 20 °C/min, and hold for 3.5 min, with a total run of 25 min. The compounds were detected by a mass spectrometer operating at 70 eV, by electron ionization. All chemicals used in this procedure were obtained from MilliporeSigma, USA.

### 3.7. Glycosidic Linkages Determination by Methylation Analysis

CB-EPS was per-*O*-methylated using the modified method of Ciucanu and Kerek (1984). An aliquot (10 mg) was solubilized in dimethylsulfoxide (1 mL), at 40 °C and after cooling, 1 mL of iodomethane and powdered NaOH (20 mg) were added to the tube [45]. The mixture was stirred for 30 min or until it turned to a solid phase, which was left to react for 14–18 h. The material was re-solubilized in water, on ice, and neutralized with glacial acetic acid. After dialysis (3.5 kDa) to remove the excess salts, the partially *O*-methylated polysaccharides were freeze-dried, and the methylation procedure was repeated to guarantee complete methylation of the free hydroxyls. The sample was then partitioned between chloroform (1 mL) and distilled water (3 mL, 3×). The chloroform phase, containing the per-*O*-methylated derivatives, was evaporated, and submitted to hydrolysis as follows: methanolysis with 3% MeOH-HCl (1 mL), for 2 h, at 80 °C, followed by evaporation under N_2_. The material was then submitted to hydrolysis with 1 M H_2_SO_4_ (1 mL), for 13 h, at 100 °C. Afterward, the sample was reduced with NaB^2^H_4_ and acetylated as above (Section 3.6), to give a mixture of partially *O*-methylated alditol acetates, which were analyzed by GC–MS using the same column as described in Section 3.6. A few modifications to the method were necessary: the flow rate was 1.46 mL/min, and the oven was programmed with a non-linear heat ramp, starting at 100 °C and holding for 1 min, increasing to 150 °C at a range of 20 °C/min, and then reaching to 200 °C at a range of 5 °C/min, being held for 5.5 min. The final temperature was set at 250 °C, heated at 25 °C/min, and hold for 5 min, with a total run of 26 min. All chemicals used in this procedure were obtained from MilliporeSigma, USA.

The derivatives were identified from *m*/*z* of their positive ions, by comparison with standards, and the results were expressed as a relative percentage of each component [46].

### 3.8. Determination of Homogeneity of CB-EPS and Molar Mass

The analysis was performed in a high-performance size-exclusion chromatography (HPSEC) coupled to a refractive index detector. An aqueous NaNO_2_ (0.1 M) containing aqueous NaN_3_ (200 ppm) was used as an eluent at a flow rate of 0.6 mL/min. The sample (1 mg/mL) was solubilized in this solution, filtered through a cellulose membrane (0.22 μm), and injected (100 μL loop) into HPSEC. The relative molar mass of the polymer was estimated using Astra software at 4.70. The reagents for preparing the solvents were obtained from MilliporeSigma, USA.

### 3.9. Spectroscopy Analyses

The NMR experiments (Heteronuclear Single Quantum Correlation—Distortionless Enhancement by Polarization Transfer: HSQC-DEPT) were obtained using a 400 MHz Bruker spectrometer model Avance III with a 5 mm inverse probe. CB-EPS (50 mg) was dissolved in Me_2_SO-*d*_6_ and the analysis was performed at 70 °C. Chemical shifts (δ) were expressed in ppm relative to the solvent ^13^C (δ 39.7) and ^1^H (δ 2.40) resonances. The NMR chemical shifts were assigned according to HSQC-DEPT experiments and literature data.

FT-IR analysis was performed in a Vertex 70 spectrometer (Bruker, Germany) equipped with an attenuated total reflectance (ATR) sampling module and a germanium crystal internal reflection element. The EPS was measured in direct contact with the ATR Ge crystal plate. Data collection was completed using the OPUS 7 software package. The spectrum was acquired with 16 scans between 4000 and 400 cm^−1^ at a resolution of 4 cm^−1^.

### 3.10. Macrophage Isolation

Albino Swiss mice (6–8 weeks old) were used as peritoneal macrophage donors. This study used all legal recommendations of Brazilian legislation (Law No. 11.794/Oct. 2008) for animal handling procedures for scientific research was used, and the Animal Ethics Committee of Unioeste approved this study. The mouse peritoneal macrophages were collected by infusing the donors’ peritoneal cavity with 8–10 mL of chilled PBS. Then, the cells were plated in the culture medium (Roswell Park Memorial Institute-RPMI 1640, 5% fetal bovine serum and antibiotics, all obtained from MilliporeSigma, USA) in 24- or 96-well plates. After 1–2 h incubation at 37 °C under 5% CO_2_ in a humidified incubator, non-adherent cells were removed by washing twice with PBS at 37 °C [47].

### 3.11. Effect of CB-EPS on Peritoneal Macrophages Cell Viability Assay

Adherent macrophages (2 × 10^5^ cells/well) were incubated for 48 h with the RPMI medium in the absence (control) or presence of varying CB-EPS concentrations (0.1 to 5.0 μg/mL). Cytotoxicity was evaluated using the MTT reagent, as described by Reilly et al. (1998) [48].

### 3.12. Measurement of Nitric Oxide (NO) Production by CB-EPS Macrophage-Treated

Peritoneal macrophages (2 × 10^5^ cells/well) were seeded in a 96-well plate and incubated in the absence (control) presence of CB-EPS (0.1, 0.25, and 0.5 μg/mL). Positive control with Lipopolysaccharide (LPS) (Sigma™) (50 ng/mL) was performed. After 48 h, the NO production was indirectly assessed by measuring nitrite concentrations in the culture medium using the Griess reaction [49] with modifications. The isolated supernatants were mixed with equal volumes of Griess reagent and incubated at 25 °C for 10 min when absorbance was measured at 550 nm in a microplate reader. The nitrite concentration was calculated from a standard NaNO_2_ curve (5–100 μM), and the results were expressed as μmol per 2 × 10^5^ cells. LPS contamination of CB-EPS was completed as described by Santana-Filho et al., and no contamination was detected [50].

### 3.13. Measurement of Superoxide Production by CB-EPS Macrophage-Treated

To determine superoxide production, macrophages were incubated for two hours in a 96-well plate with a standard reaction mixture consisting of HBSS containing nitroblue tetrazolium (NBT; MilliporeSigma, USA) (0.025%) in the presence of CB-EPS (0.1, 0.25, and 50 μg/mL). In addition, Phorbol 12-myristate 13-acetate (PMA 1 µg/mL; MilliporeSigma, USA) was prepared in non-toxic amounts of DMSO and used as the positive control. Absorbance was measured at 550 nm, and the amount of superoxide anion released was demonstrated as previously shown [51]. The results were expressed in percentages, with the control as 100%.

### 3.14. Superoxide Anion Scavenging Activity

The scavenging ability of superoxide radicals was assessed by the method described by Costa et al. [52]. The CB-EPS was diluted (1, 2.5, and 5 μg/mL) in purified water and added to the reaction mixture, which consisted of 10 mM Tris–HCl buffer pH 8.0, 338 μM NADH, 72 μM NBT, and 30 μM PMS. The positive control consisted only of NADH and NBT, i.e., a control that does not generate anions, and the control consisted of NADH, NBT, and PMS, which generates superoxide anions. The reaction was monitored at 560 nm for 5 min, and the scavenging ability of superoxide radicals was calculated using the following Equation (2):

Scavenging ability % = {1 − (Abs sample/Abs control)} × 100(2)

### 3.15. Quantification of IL-6, IL-10, and TNF-α Production by CB-EPS Macrophage-Treated

Peritoneal macrophages (1 × 10^6^ cells/well) were plated on a 24-well plate with RPMI medium in the absence (negative control) or presence of varying CB-EPS concentrations (0.01, 0.025, and 0.05 μg/mL) for 6 h (TNF-α) and 48 h (IL-6 and IL-10) at 37 °C under 5% CO_2_. LPS (50 ng/mL) was used as the positive control, and the supernatant from each well was collected and maintained at −80 °C until utilization. IL-6, IL-10, and TNF-α concentrations in the supernatant were determined using an enzyme-linked immunosorbent assay (ELISA) specified by the manufacturers (Peprotech Inc.™, Westlake Village, CA, USA). The results are expressed in pg/mL or ng/mL. No components of the culture medium showed immunoreactivity to the cytokines under study.

### 3.16. Assay for Phagocytic Activity

Phagocytic activity was assayed as described by Melo et al. using zymosan as the phagocytosing particle [53]. Peritoneal macrophages (2 × 10^5^ cells/well), adhered onto a tissue culture plate (96 wells), were incubated with the RPMI medium in the absence (control) or presence of CB-EPS (0.10, 0.25, and 0.50 μg/mL). After 48 h at 37 °C under 5% CO_2,_ the cells were washed three times with RPMI medium, and zymosan (1 × 10^8^ particles/mL; MilliporeSigma, USA), previously incubated with neutral red, was added following incubation under the same conditions for 30 min. After incubation, non-phagocytosed particles were removed by rinsing with PBS. The cells were then fixed with Baker’s fixative and lysed with a hydroalcoholic solution with 2% glacial acetic acid. The optical densities of the phagocytosed particles were read at 550 nm and compared to the absence (100% phagocytosis) and presence of CB-EPS. The results are expressed as relative phagocytic activity.

### 3.17. Statistical Analysis

Optimization of the CB-EPS production was evaluated by Statistica 7.0 software, using the ANOVA method. In addition, the data from biological activity were analyzed statistically by ANOVA and Tukey’s post-test by GraphPad Prism 6.0 Software.

## 4. Conclusions

*Curvularia brachyspora*, a black mold with essential biotech capabilities, was evaluated in this study to determine the best conditions to produce exopolysaccharides. RSM was used for the optimization process, which yielded 75.05% of total sugar in the optimized conditions. The chemical analyses showed the presence of exopolysaccharides, and although they were characterized as a polydisperse polymer, showing a non-uniform peak, it was possible to observe signals (NMR) and derivatives (methylation analysis) relative to a β-d-glucan and a highly branched glucogalactomannan. Such molecules induced macrophages to produce cytokines but did not stimulate the liberation of ROS/NO, indicating an indirect antimicrobial activity. Many fungal polysaccharides induce an effect on immune cells. This response was confirmed for CB-EPS, demonstrating an application of such exopolysaccharides that could be cultivated in large amounts using the optimized conditions determined in the present study. A profound investigation into CB-EPS biological effects should be employed to provide more details for their applicability.

## Figures and Tables

**Table 1:** Experimental matrix design (real and coded values) of DCCR experiment and the corresponding results in the exopolysaccharide production by *C. brachyspora*.

Run	X_1_ (pH)	X_2_ (Time hs)	X_3_ (Urea %)	% Total Sugar
1	−1 (7.4)	−1 (139)	−1 (0.1)	29.56
2	+1 (8.6)	−1 (139)	−1 (0.1)	21.60
3	−1 (7.4)	+1 (197)	−1 (0.1)	75.05
4	+1 (8.6)	+1 (197)	−1 (0.1)	27.54
5	−1 (7.4)	−1 (139)	+1 (0.35)	20.37
6	+1 (8.6)	−1 (139)	+1 (0.35)	31.08
7	−1 (7.4)	+1 (197)	+1 (0.35)	36.38
8	+1 (8.6)	+1 (197)	+1 (0.35)	29.09
9	−1.68 (7)	0 (168)	0 (0.25)	36.31
10	+1.68 (9)	0 (168)	0 (0.25)	7.26
11	0 (8)	1.68 (120)	0 (0.25)	20.55
12	0 (8)	+1.68 (216)	0 (0.25)	44.84
13	0 (8)	0 (168)	−1.68 (0)	24.8
14	0 (8)	0 (168)	+1.68 (0.5)	17.60
15	0 (8)	0 (168)	0 (0.25)	30.96
16	0 (8)	0 (168)	0 (0.25)	32.11
17	0 (8)	0 (168)	0 (0.25)	33.13

**Table 2:** Model coefficients estimated by multiple linear regression (significance of regression coefficients).

	SS	df	MS	*p* *	F
x1 (pH)	855.915	1	855.9147	0.004003	17.69460
$x_{1}^{2}$	42.827	1	42.8268	0.378063	0.88537
x2 (Time (hs))	900.433	1	900.4330	0.003504	18.61495
$x_{2}^{2}$	39.915	1	39.9150	0.393870	0.82518
x3 (Urea (%))	158.375	1	158.3753	0.113304	3.27414
$x_{3}^{2}$	61.190	1	61.1898	0.297790	1.26500
x1x2	414.000	1	414.0003	0.022165	8.55876
x1x3	405.790	1	405.7904	0.023105	8.38904
x2x3	177.226	1	177.2262	0.097167	3.66385
Error	338.601	7	48.3715		
Total SS	3300.182	16			

* Significant results are in red.

**Table 3:** Partially *O*-methylalditol acetates formed on methylation reaction of CB-EPS produced by *C. brachyspora*.

Partially *O*-Methylated Alditol Acetates ^a^	Rt (min) ^b^	Mol %	Linkage Type ^c^
2,3,4,6-Me_4_-Glc*p*	13.335	38.0	Glc*p*-(1→
2,3,5,6-Me_4_-Gal*f*	13.475	0.6	Gal*f*-(1→
2,3,4,6-Me_4_-Gal*p*	13.715	0.9	Gal*p*-(1→
3,4,6-Me_3_-Man*p*	14.910	8.7	2→)-Man*p*-(1→
2,4,6-Me_3_-Glc*p*	14.975	3.7	3→)-Glc*p*-(1→
2,3,6-Me_3_-Hex	15.030	1.8	4→)-Hex-(1→
2,3,6-Me_3_-Gal*f*	15.170	8.6	5→)-Gal*f*-(1→
2,3,4-Me_3_-Man*p*	15.495	15.7	6→)-Man*p*-(1→
2,3,5-Me_3_-Gal*f*	15.925	3.7	6→)-Gal*f*-(1→
3,4-Me_2_-Man*p*	17.295	6.3	2,6→)-Man*p*-(1→
2,4-Me_2_-Glc*p*	17.580	3.1	3,6→)-Glc*p*-(1→
3,5-Me_2_-Gal*f*	17.940	8.9	2,6→)-Gal*f*-(1→

^a^ Analyzed by GC-MS after methylation, total acid hydrolysis, reduction with NaBD_4_, and acetylation; ^b^ Retention time; ^c^ Based on derived *O*-methylalditol acetates.

## Data Availability

The data presented in this study are available on request from the corresponding authors.

## Supplementary Materials

The following supporting information can be downloaded at: https://www.mdpi.com/article/10.3390/molecules28114356/s1, Figure S1: Kinetic of glucose consumption in the medium and production of EPS by *Curvularia brachyspora*. The values shown are the mean of two experiments in triplicate, and the fungus *C. brachyspora* was cultured at optimized conditions (see main text); Figure S2: Partially alditol acetates formed on acetylation analysis of CB-EPS (panel A), analyzed by GC–MS, after total acid hydrolysis, reduction with NaBH4 and acetylation. The monosaccharides were identified according the m/z of their positive ions (Panel C), and the hexoses (mannose, galactose, and glucose) were differentiated according to their retention time in comparison to standards (Panel B); Table S1: Experimental matrix design (real and coded values) of 2^4^ factorial experiments, using as variables pH, time (days), temperature (°C), and shaking (RPM); Table S2: Experimental matrix design (real and coded values) of 2^6−1^ factorial experiment, using as variables glucose concentration (%), pH, time (days), Ammonium Nitrate (%), Urea (%), and Sodium Nitrate (%).
