# Supplementary material for: Biotech Application of Exopolysaccharides from Curvularia brachyspora: Optimization of Production, Structural Characterization, and Biological Activity
Source: Molecules. 2023 May 26;28(11):4356. doi: 10.3390/molecules28114356 (PMC10254318; doi:10.3390/molecules28114356)
Supplement: Supplementary file 1 [file molecules-28-04356-s001.zip › molecules-2333747-supplementary.pdf]

Supplementary material

Figure S1 – Kinetic of glucose consumption in the medium and production of EPS by Curvularia brachyspora. The values shown are the mean of two experiments in triplicate, and the fungus C. brachyspora was cultured at optimized conditions (see main text).

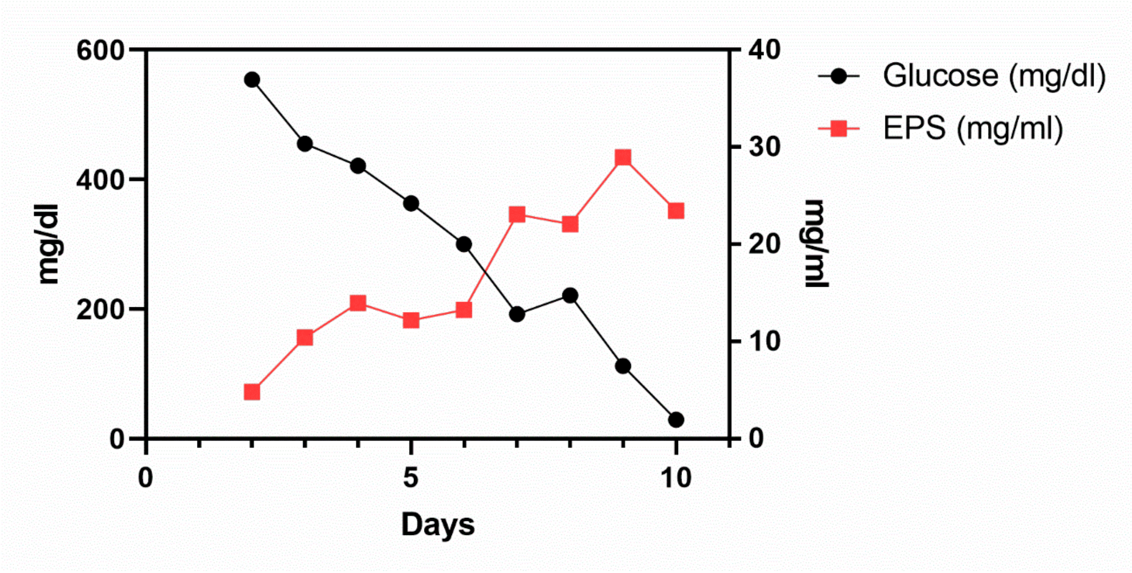

Figure S2 - Partially alditol acetates formed on acetylation analysis of CB-EPS (panel A), analyzed by GC–MS, after total acid hydrolysis, reduction with NaBH4 and acetylation. The monosaccharides were identified according the m/z of their positive ions (Panel C), and the hexoses (mannose, galactose, and glucose) were differentiated according to their retention time in comparison to standards (Panel B).

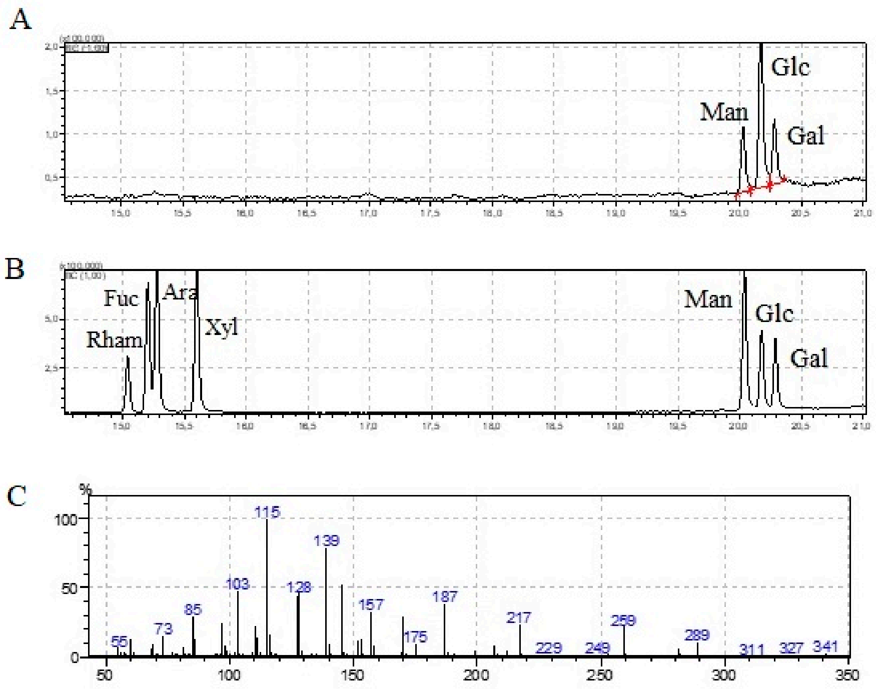

Table S1 - Experimental matrix design (real and coded values) of 2^4 factorial experiments, using as variables pH, time (days), temperature (ºC), and shaking (RPM).

Run	pH	Time (days)	Temperature (ºC)	Shaking (RPM)
1	-1 (4)	-1 (2)	-1 (25)	-1 (0)
2	+1 (10)	-1 (2)	-1 (25)	-1 (0)
3	-1 (4)	+1 (10)	-1 (25)	-1 (0)
4	+1 (10)	+1 (10)	-1 (25)	-1 (0)
5	-1 (4)	-1 (2)	+1 (40)	-1 (0)
6	+1 (10)	-1 (2)	+1 (40)	-1 (0)
7	-1 (4)	+1 (10)	+1 (40)	-1 (0)
8	+1 (10)	+1 (10)	+1 (40)	-1 (0)
9	-1 (4)	-1 (2)	-1 (25)	+1 (120)
10	+1 (10)	-1 (2)	-1 (25)	+1 (120)
11	-1 (4)	+1 (10)	-1 (25)	+1 (120)
12	+1 (10)	+1 (10)	-1 (25)	+1 (120)
13	-1 (4)	-1 (2)	+1 (40)	+1 (120)
14	+1 (10)	-1 (2)	+1 (40)	+1 (120)
15	-1 (4)	+1 (10)	+1 (40)	+1 (120)
16	+1 (10)	+1 (10)	+1 (40)	+1 (120)
17	0 (7)	0 (6)	0 (32.5)	0 (60)
18	0 (7)	0 (6)	0 (32.5)	0 (60)
19	0 (7)	0 (6)	0 (32.5)	0 (60)

Table S2 - Experimental matrix design (real and coded values) of 2^{6-1} factorial experiment, using as variables glucose concentration (%), pH, time (days), Ammonium Nitrate (%), Urea (%), and Sodium Nitrate (%).

Run	pH	Time (days)	Glucose (%)	Ammonium Nitrate (%)	Sodium Nitrate (%)	Urea (%)
1	-1 (4)	-1 (2)	-1 (25)	-1 (0)	-1 (0)	-1 (0)
2	+1 (10)	-1 (2)	-1 (25)	-1 (0)	-1 (0)	+1 (2)
3	-1 (4)	+1 (10)	-1 (25)	-1 (0)	-1 (0)	+1 (2)
4	+1 (10)	+1 (10)	-1 (25)	-1 (0)	-1 (0)	-1 (0)
5	-1 (4)	-1 (2)	+1 (40)	-1 (0)	-1 (0)	+1 (2)
6	+1 (10)	-1 (2)	+1 (40)	-1 (0)	-1 (0)	-1 (0)
7	-1 (4)	+1 (10)	+1 (40)	-1 (0)	-1 (0)	-1 (0)
8	+1 (10)	+1 (10)	+1 (40)	-1 (0)	-1 (0)	+1 (2)
9	-1 (4)	-1 (2)	-1 (25)	+1 (2)	-1 (0)	+1 (2)
10	+1 (10)	-1 (2)	-1 (25)	+1 (2)	-1 (0)	-1 (0)
11	-1 (4)	+1 (10)	-1 (25)	+1 (2)	-1 (0)	-1 (0)
12	+1 (10)	+1 (10)	-1 (25)	+1 (2)	-1 (0)	+1 (2)
13	-1 (4)	-1 (2)	+1 (40)	+1 (2)	-1 (0)	-1 (0)
14	+1 (10)	-1 (2)	+1 (40)	+1 (2)	-1 (0)	+1 (2)
15	-1 (4)	+1 (10)	+1 (40)	+1 (2)	-1 (0)	+1 (2)
16	+1 (10)	+1 (10)	+1 (40)	+1 (2)	-1 (0)	-1 (0)
17	-1 (4)	-1 (2)	-1 (25)	-1 (0)	+1 (2)	+1 (2)
18	+1 (10)	-1 (2)	-1 (25)	-1 (0)	+1 (2)	-1 (0)
19	-1 (4)	+1 (10)	-1 (25)	-1 (0)	+1 (2)	-1 (0)

20	+1 (10)	+1 (10)	-1 (25)	-1 (0)	+1 (2)	+1 (2)
21	-1 (4)	-1 (2)	+1 (40)	-1 (0)	+1 (2)	-1 (0)
22	+1 (10)	-1 (2)	+1 (40)	-1 (0)	+1 (2)	+1 (2)
23	-1 (4)	+1 (10)	+1 (40)	-1 (0)	+1 (2)	+1 (2)
24	+1 (10)	+1 (10)	+1 (40)	-1 (0)	+1 (2)	-1 (0)
25	-1 (4)	-1 (2)	-1 (25)	+1 (2)	+1 (2)	-1 (0)
26	+1 (10)	-1 (2)	-1 (25)	+1 (2)	+1 (2)	+1 (2)
27	-1 (4)	+1 (10)	-1 (25)	+1 (2)	+1 (2)	+1 (2)
28	+1 (10)	+1 (10)	-1 (25)	+1 (2)	+1 (2)	-1 (0)
29	-1 (4)	-1 (2)	+1 (40)	+1 (2)	+1 (2)	+1 (2)
30	+1 (10)	-1 (2)	+1 (40)	+1 (2)	+1 (2)	-1 (0)
31	-1 (4)	+1 (10)	+1 (40)	+1 (2)	+1 (2)	-1 (0)
32	+1 (10)	+1 (10)	+1 (40)	+1 (2)	+1 (2)	+1 (2)
33	0 (7)	0 (6)	0 (32.5)	0 (1)	0 (1)	0 (1)
34	0 (7)	0 (6)	0 (32.5)	0 (1)	0 (1)	0 (1)
35	0 (7)	0 (6)	0 (32.5)	0 (1)	0 (1)	0 (1)
